# The Effect of Problem Construction on Team Process and Creativity

**DOI:** 10.3389/fpsyg.2018.02098

**Published:** 2018-11-05

**Authors:** Roni Reiter-Palmon, Vignesh Murugavel

**Affiliations:** Department of Psychology, University of Nebraska Omaha, Omaha, NE, United States

**Keywords:** teams, creativity, creative process, problem construction, creative problem solving

## Abstract

Although research on the benefits of problem construction within the creative process is expanding, research on team problem construction is limited. This study investigates the cognitive process of problem construction and identification at the team level through an experimental design. Furthermore, this study explores team social processes in relation to problem construction instructions. Using student teams solving a real-world problem, the results of this study revealed that teams that engaged in problem construction and identification generated more original ideas than teams that did not engage in such processes. Moreover, higher satisfaction and lower conflict was observed among groups that engaged in problem construction compared to groups that did not engage in problem construction. These findings highlight the utility of problem construction for teams engaging in creative problem-solving.

## Introduction

In the last two decades, interest in creativity and innovation has grown tremendously. Creativity and innovation have been suggested as important for organizational performance (Dess and Picken, 2000; Shalley et al., 2004; Mumford and Hunter, 2005). In addition, increased frequency and rapid changes in technology, globalization, and increased competition have all created an environment in which creativity and innovation are necessary for organizational survival (Mumford et al., 2002b; Shalley et al., 2004). Specifically, creativity has been defined in terms of the production of a “novel product, idea, or problem solution that is of value to the individual and/or the larger social group” (Hennessey and Amabile, 2010, p. 572). The implementation of a creativity idea or solution and application of a creative product is referred to as innovation (Amabile, 1988).

Interest and research on team creativity has increased in recent years as a result of the complexity of problems that face organization exceeding the capabilities of any single individual (Shalley et al., 2004; Kozlowski and Bell, 2008; Reiter-Palmon et al., 2012). In the past, team creativity research has focused on evaluating the role of the creative individual as part of the team (Taggar, 2002). This research identified the relationship between specific team variables such as team diversity, team social processes such as conflict, and social cognitive processes such as shared mental models to creativity exhibited by each individual within the team (Hulsheger et al., 2009). However, less research has directly evaluated the factors that influence team creativity as a construct, as opposed to individual creativity within the team context (Reiter-Palmon et al., 2008).

Creative problem solving is an aspect of creativity that has been researched extensively at the individual level (Merrifield et al., 1962; Basadur, 1982; Silverman, 1985; Sternberg, 1988; Mumford et al., 1991; Finke et al., 1992). While the specific phases and stages of these models differ to some extent, all of these models suggest that creative problem solving starts with problem identification and construction, followed by idea generation, then idea evaluation and selection (Mumford et al., 1991; Reiter-Palmon, 2018). The problem construction process is of particular importance due to the nature of the problems that allow for or require creativity. Problems that allow for creative solutions tend to be novel, ambiguous and ill-defined (Schraw et al., 1995). Ill-defined problems are characterized by multiple possible goals, multiple possible approaches to solving the problem, and multiple possible and acceptable solutions (Mumford et al., 1991; Schraw et al., 1995). Idea generation or brainstorming focuses on the development of ideas or solutions to the problem, and has been the focus of much of the research on creativity (Reiter-Palmon et al., 2008). Finally, ideas are evaluated to determine which of the ideas should be implemented (Mumford et al., 2002a).

## Team Cognitive Processes: Problem Construction

Problem identification and construction refers to the process in which a problem is identified by the problem solver, an ill-defined problem is structured, and the parameters of that problem are defined (Reiter-Palmon and Robinson, 2009). Problem construction allows individuals to develop and provide some structure and direction to an ambiguous, ill-defined problem. At the individual level, creative individuals have been shown to engage in the process more so than their less creative counterparts (Getzels and Csikszentmihalyi, 1975; Rostan, 1994). However, it has been suggested that for most individuals the process of problem construction occurs automatically, and individuals are not aware that they are defining a problem (Mumford et al., 1994). Past research has demonstrated that active engagement in problem construction, through the use of instructions, has increased the creativity of the solutions developed and that the quality and originality of how the problem is constructed is directly related to quality and originality of the solutions generated (Mumford et al., 1994; Reiter-Palmon et al., 1997; Arreola and Reiter-Palmon, 2016). Because problem construction provides structure and allows individuals to manage and organize an ambiguous, ill-defined problem, it is not surprising that problem construction has been found to have a significant effect on creative problem solving (Ma, 2009).

However, research evaluating this process in teams is sparse. Reiter-Palmon et al. (2008) suggested that it is likely that teams focus on discussing solutions rather than discussing various problem constructions. Consequently, individuals are not aware of how they construct the problem, and potential differences in how different individuals within the team understand and define the problem. Further, it has been suggested that conflict regarding solutions may be rooted in differences in how problems are structured and goals are understood (Cronin and Weingart, 2007; Reiter-Palmon et al., 2008) Research indicates that creative teams suffer when problem frameworks vary across team members, and the goal states identified through problem construction cannot be reconciled in a single solution (Cronin and Weingart, 2007; Goh et al., 2013). Cronin and Weingart (2007) refer to these differences as *representational gap* or rGaps. Teams with larger rGaps tend to have difficulty during problem construction, leading to poor cognitive integration as a team and lower creativity (Weingart et al., 2005). However, research has also suggested that larger rGaps may increase team creativity when teams identify the discrepancies early and use them to communicate about alternative pathways to solving the problem (Weingart et al., 2008). Differences in cognitive representation among group members have also been linked to team processes beyond problem construction. Cronin et al. (2011) found that these differences affect the formation of subgroups within a team, which can lead to potentially negative outcomes such as a decrease in satisfaction or effectiveness.

Research has also supported the notion that individuals rely on education and past experiences when developing an understanding of the problem, and therefore team members may construct problems differently. Leonardi (2011) found that individuals from different departments structured and constructed problems differently; however, they were largely unaware that they had different ways of conceptualizing the problem. Leonardi further found that leaders were especially important in resolving these differences, such that when leaders encouraged teams to discuss problem features they were able to develop a shared framework or construction. This mutually understood structure in turn guided the innovation process. Similarly, Gish and Clausen (2013) found that prior knowledge influenced how individuals within teams constructed problems. These teams also suffered from team conflict and disagreements during idea generation and team members were unaware of these differences in problem constructions. This conflict, in turn, resulted in lowered creativity. However, when additional information that facilitated divergence in problem construction to identify multiple problem definitions was introduced, teams were more effective at generating an innovative solution.

The current limited research on problem construction in teams suggests that differences in how individuals think about the problem are related to conflict, and that when this conflict is not resolved, creativity suffers (Weingart et al., 2005, 2008; Leonardi, 2011; Gish and Clausen, 2013). The studies discussed all imply that team processes such as team conflict, directly influence the creative processes. In addition, the research described above was all conducted in natural settings with no experimental controls. It is therefore difficult to determine whether conflict was a result of differences in problem construction, was the cause of differences, or whether conflict and create processes co-ocurred. Other work on social processes and facets of creative problem solving suggest that the social processes of psychological safety and conflict may limit the effectiveness of cognitive processes such as information sharing or information elaboration (Hoever et al., 2012; Qu and Liu, 2017). Similarly, the Motivated Information Processing in Groups (MIP-G) framework suggests that effective and deep information processing or cognition in teams that leads to team creativity will occur when social processes are effective (De Dreu et al., 2011). Supporting evidence to the effect of poor social processes such as conflict and low trust comes from work on team diversity and its effect on team creativity. Leung and Wang (2015) found that poor team social processes, resulting from team diversity, hinder knowledge sharing and communication, which in turn result in lowered team creativity. Further, Cronin et al. (2011) suggest that for the group to take advantage of different points of view and the richness of information that is available to different individuals, team members must share that information. They further suggest that cognitive integration becomes more difficult when there are different subgroups within the team.

Research and theory to date have focused on the role of social processes and their effect on team cognition or how the two occur concurrently. That is, studies have suggested that poor communication, conflict, low trust and other less effective social processes *resulted in* less effective cognition and therefore reduced creativity. While social processes can have an effect on cognitive processes, the reverse question, of whether cognitive processes, such as problem construction, can have an effect on social process, has not been addressed. As the process of problem construction aims to provide structure to an ambiguous problem, team engagement in the process may facilitate information sharing and discussion, allowing for better communication and sharing of ideas. As the research described above suggests, team members that construct the problem differently may not be aware of these differences (Weingart et al., 2005, 2008; Leonardi, 2011; Gish and Clausen, 2013). We therefore expect that active engagement in the problem construction process may facilitate understanding of the different ways in which team members understand the problem, and therefore can also influence the effectiveness of social processes in teams.

## Current Study

Before understanding how active engagement in problem construction processes influence team social processes, however, it was important to determine how active engagement was to be manipulated at the team level. At the individual level, this is accomplished by asking the individual to restate the problem in many different ways, prior to solving the problem. At the team level, these instructions could be given to individuals or to the team as a whole. At this point, the theoretical models of individual or team cognition do not specify which approach may be best, or how these approaches may differ (Reiter-Palmon et al., 2008; Reiter-Palmon, 2018). As a result, it is possible that variations in the instructions to teams may influence the effectiveness of such instructions. Past research on instructions (focusing on divergent thinking tasks) has found that specific instructions can result in specific effects such that instructions to generate multiple ideas result in more ideas generated, whereas instructions to generate original ideas result in more original ideas being generated. Specifically, instructions can be given to individuals, facilitating problem construction at the individual level, but may or may not result in team discussion about problem construction. Instructions can be given at the team level, resulting in team discussion, but potentially limiting individual problem construction. Finally, instructions can be focused on both individual and then team problem construction, potentially maximizing both. Therefore, the first aim of the study was to directly compare three different approaches to manipulate active engagement in team problem construction to determine whether they are equivalent or whether they result in different outcomes related to solution creativity.

The second aim was to determine whether explicitly engaging problem construction prior to solving a problem in a team context would result in increased solution quality and originality, replicating individual level findings. Finally, the third aim of the study was to determine whether engagement in problem construction influenced any team processes, particularly those that relate to conflict and satisfaction. As it has been speculated that difficulties in team social processes such as conflict may arise due to differences in how problems are constructed, and that team members may not realize that they are constructing problems in dissimilar ways, it was expected that instructions to engage in problem construction would result in less conflict and increased satisfaction.

## Materials and Methods

### Participants

The study was conducted using 65 groups. Each group consisted of three individuals who signed up for the study in the same timeslot. If more than three participants were signed up, they were randomly assigned to groups. If only three participants were signed up, they comprised the group. The total number of participants was 195, of which 109 were female (57.1%) and 82 were male (42.9%), with participants not responding. Average age was 22.88 (sd = 6.26). Groups were randomly assigned to one of four conditions.

### Procedure

In all conditions, groups were presented and asked to solve a real-life problem relevant to students in which a student is having trouble with his current academic and extracurricular workload. Groups are asked to provide a solution to the student about his plans for the upcoming semester. The first condition was a control condition in which the team did not engage in problem construction. The group only provided a solution to the problem. The other three conditions varied on their problem construction manipulations.

#### Problem Construction Manipulation

As problem construction has not previously been manipulated in a team setting, three different conditions were used. Manipulations differed in the instructions given to the participants, and whether the focus was on individual generation or team generation of problem constructions. The purpose of including the three conditions was to determine whether there were any differences in the effectiveness of these instructions. In the first manipulation of problem construction, participants were asked to generate as many restatements of the problem as they could individually before proceeding to solve the problem as a team. In the second manipulation, participants engaged in both problem construction and solution generation as a team. Finally, in the third manipulation, participants were instructed to generate as many restatements as they could to the problem individually, then reach consensus on these as a team, and then move on to developing a solution. Once the team completed the solution generation task, participants completed a number of measures including satisfaction with the team process and team outcome, a measure of team conflict, and demographics.

### Measures

#### Team conflict

Conflict within the groups was measured using Jehn and Mannix (2001) nine-item scale. The scale contains three subscales of intragroup conflict. The first subscale pertains to task conflict (i.e., “How much conflict of ideas is there in your work group?”; α = 0.94). The second subscale involves relationship conflict (i.e., “How much relationship tension is there in your work group?”; α = 0.94). The third subscale relates to process conflict (i.e., “How often are there disagreements about who should do what in your work group?”; α = 0.93). Group members indicated the degree to which they experienced what was on each item using a Likert-style scale ranging from 1 = *none* to 5 = *a great deal.*

#### Team Satisfaction

Satisfaction with the team processes and outcomes was measured using two subscales of a group satisfaction scale developed by Briggs et al. (2006). Participants indicated their degree of agreement with statements on a seven-point Likert scale ranging from 1 = *strongly disagree* to 7 = *strongly agree*. Items from the subscale of the team processes pertained to feeling of satisfaction with procedures and operations followed by the group (e.g., “I feel satisfied with the procedures used in today’s meeting”). A Cronbach’s alpha of 0.96 was observed for this subscale. Items from the team outcomes subscale involved feelings of satisfaction related to the achievements of the group (e.g., “When the meeting was finally over, I felt satisfied with the results”; α = 0.93).

#### Problem Solving

Solutions were rated for creativity by trained raters using a modified Consensual Assessment Technique (Amabile, 1996). Raters were graduate and undergraduate students. Raters were also blind to the study’s conditions. Raters received extensive training which involved a review of creativity, an overview of the rating scale system, the problem used in this study, and aspects of creativity to rate. Two raters assessed originality and three raters assessed quality as aspects of creativity. Originality refers to the uniqueness of the solution, whereas quality refers to the appropriateness and viability of the solution. Both facets of creativity were evaluated on a 1 = *very low* to 5 = *very high* scale. The two raters’ scores for originality were averaged, resulting in a single originality score for each solution. The three raters’ scores for quality were also averaged, resulting in a single quality score for each solution. Interclass correlations of 0.88 among ratings of originality and 0.94 for quality ratings were observed, indicating acceptable rater agreement (Shrout and Fleiss, 1979).

## Results

To address the methodological issue of which instructions for problem construction are effective in terms of their effect on solution quality and originality, the three conditions of problem construction were compared. One-way ANOVAs were conducted to compare the three problem construction conditions on originality and quality separately. No group differences in originality, *F*(2,43) = 0.56, *p* = 0.578, or quality, *F*(2,43) = 0.68, *p* = 0.513, were found based on the instructions for problem construction. Therefore, the three conditions were collapsed into one condition, allowing for control group to general problem construction manipulation comparisons. As a result, the following analysis reflected 19 groups in the control condition and 46 groups in the problem construction condition.

**FIGURE 1:**
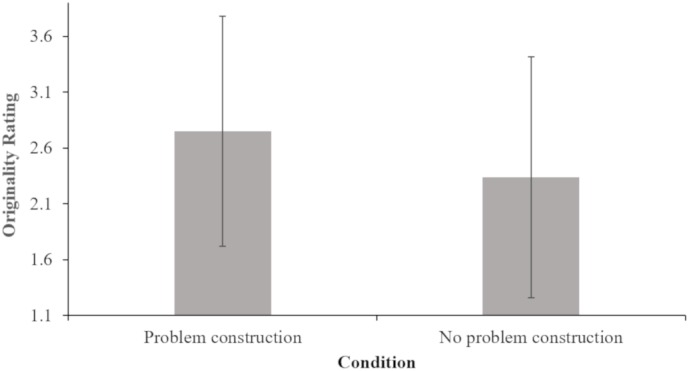
Mean originality ratings of solutions from problem construction and no problem construction instruction conditions.

The second set of analyses was conducted to determine whether differences exist between teams that were asked to construct the problem and teams that were not asked in terms of the originality and quality of the solutions generated. Two ANOVAs were conducted to compare solution originality and quality, respectively, in problem construction and no problem construction conditions. Results indicated that there were marginal differences in solution originality for the problem construction condition and no problem construction condition *F*(1,63) = 2.06, *p* = 0.078; *eta squared* = 0.03), see Table 1 and Figure 1. Teams that engaged in problem construction generated marginally significantly more original solutions compared to those that did not engage in problem construction. There were no differences in solution quality for the problem construction condition and no problem construction condition *F*(1,65) = 0.272, *p* = 0.302. The quality of solutions from teams that engaged in problem construction did not differ from the quality of solutions from teams that did not engage in problem construction.

**Table 1:** ANOVA results comparing problem construction and no problem construction groups on solution quality and originality ratings.

		Sum of Squares	*df*	Mean Square	*F*	*p*	partial η^2^	
Quality	Contrast	0.33	1	0.33	0.27	0.304	0.00	
	Error	76.33	63	1.21				
	Total	546.92	65					
Originality	Contrast	2.24	1	2.24	2.06	0.078	0.03	
	Error	68.40	63	1.09				
	Total	520.50	65					

**FIGURE 2:**
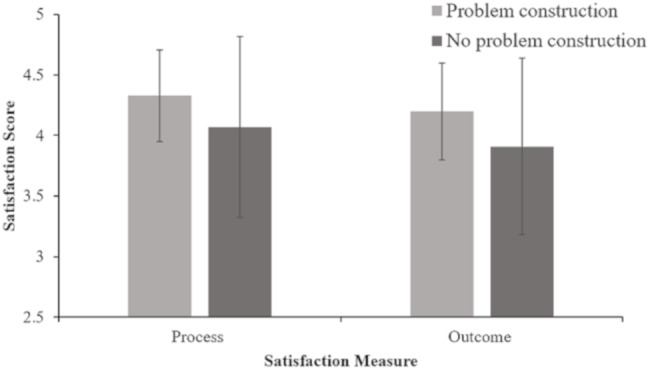
Mean satisfaction scores in problem construction and no problem construction instruction conditions.

To address the question of whether teams that engaged in problem construction were different than teams that did not engage in problem construction in terms of satisfaction and conflict, mean comparisons were used. As there were two different subscales for satisfaction and three for conflict, MANOVA was used for each one of the constructs, utilizing all the subscales. As both MANOVAs were significant, we are presenting the follow up ANOVAs on each subscale. There was a significant difference in process satisfaction between the problem construction condition and no problem construction condition *F* = 3.2, *p* = 0.040, *eta squared* = 0.05. Results for outcomes satisfaction indicated that there was a significant difference in outcome satisfaction between the problem construction condition and no problem construction condition *F* = -2.10, *p* = 0.020, *eta squared* = 0.07. That is, both process and outcome satisfaction was higher when teams engaged in problem construction compared to when teams did not engage in problem construction. See Table 2 and Figure 2.

**Table 2:** ANOVA results comparing problem construction and no problem construction groups on satisfaction measures.

		Sum of Squares	*df*	Mean Square	*F*	*p*	partial η^2^	
Process	Contrast	0.85	1	0.85	3.20	0.040	0.05	
	Error	16.68	63	0.27				
	Total	1192.30	65					
Outcome	Contrast	1.18	1	1.18	4.41	0.020	0.07	
	Error	16.78	63	0.27				
	Total	1120.19	65					

**FIGURE 3:**
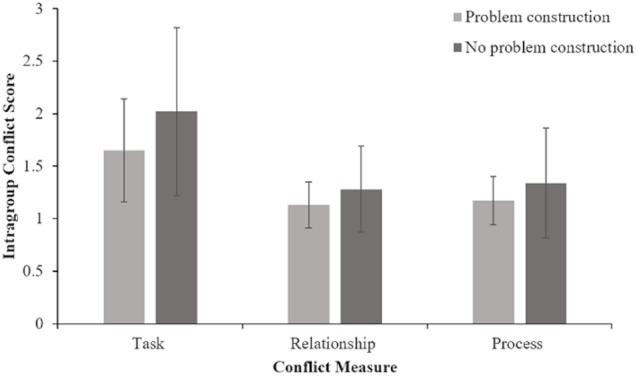
Mean intragroup conflict scores in problem construction and no problem construction instruction conditions for each conflict measure.

To evaluate whether there were differences between the problem construction condition and no problem condition for conflict, three ANOVAs were conducted. The first analysis of conflict involved task conflict. Results indicated that there was a significant difference in task conflict between the problem construction condition and no problem construction condition *F* = 5.09, *p* = 0.014, *eta squared* = 0.08. There was a significant difference in relationship conflict between the problem construction condition and no problem construction condition *F* = 3.9, *p* = 0.027, *eta squared* = 0.03. Finally, to compare process conflict in problem construction and no problem construction conditions, a third ANOVA was conducted. Results indicated that there was a significant difference in process conflict between the problem construction condition and no problem construction condition *F* = 3.21, *p* = 0.039, *eta squared* = 0.05. These results indicated that all three measures of conflict, task, outcomes, and process conflict were lower when teams engaged in problem construction compared to when teams did not engage in problem construction. See Table 3 and Figure 3.

**Table 3:** ANOVA results comparing problem construction and no problem construction group on conflict subscales.

		Sum of Squares	*df*	Mean Square	*F*	*p*	partial η^2^	
Task	Contrast	1.82	1	1.82	5.09	0.014	0.08	
	Error	22.50	63	0.36				
	Total	225.04	65					
Relationship	Contrast	0.32	1	0.32	3.90	0.027	0.06	
	Error	5.10	63	0.08				
	Total	94.74	65					
Process	Contrast	0.38	1	0.38	3.21	0.039	0.05	
	Error	7.41	63	0.12				
	Total	104.62	65					

## Discussion

This study provides the first empirical research in which team engagement in problem construction is manipulated through instructions. The findings above suggest that team problem construction can potentially benefit creativity. Although marginal, the apparent effect of problem construction on solution originality provides some initial support that team problem construction leads to creative problem solving at the team level. Limited power, as a result of a relatively small number of teams in the control condition, offers some explanation for the observed bordering significance value of originality differences between groups. Nonetheless, the role of problem construction at the team level is further elucidated through the analyses.

More importantly, team problem construction may facilitate some of the social processes that can then help in effective problem solving. Taken together, the final set of analyses show that problem construction at the team level resulted in lower conflict and higher satisfaction. Past research focused on the effect of social processes on team cognition such as information sharing and elaboration or evaluated the concurrent nature of these relationships (Hoever et al., 2012; Qu and Liu, 2017). This study, however, evaluated the effect of team cognitive processes on social processes by manipulated instructions for problem construction. This experimental design allows us to directly evaluate whether cognitive processes can have an effect on social processes. Since problem construction was a manipulated variable, and occurred prior to the measurement of social processes, the causal inference that problem construction is the cause of improved social processes is appropriate. This study, therefore, addresses the call by Reiter-Palmon et al. (2012) to further elucidate the relationships between social processes and cognitive processes. As problem construction has been suggested to provide some basic structure for creative problem solving, this reduction in conflict and increase to satisfaction might result from a reduction in the uncertainty associated with ill-defined problems (Mumford et al., 1994). Furthermore, problem construction at a team level may counter disagreement and conflict, while also promoting group satisfaction, as a result of discussions early in the process while thinking about ideas and solutions is still more malleable. This research demonstrates that this cognitive process has implications beyond the individual level, denoting the broad utility of problem construction.

Finally, it is interesting to note that there do not seem to be differences among the various instructions provided for problem construction in terms of creative performance. This lack of condition differences hints that the manner in which a team engages in problem construction may not be as important to creativity as the act of a team engaging in problem construction in and of itself.

## Limitations and Future Research

This study provides a first step in the study of manipulating problem construction in teams. One important limitation of this study was the fact that the sample size for the control group was somewhat low. This may have had a role in the marginal effect found for the originality of solutions. Future research should not only strive to replicate this research, but should include a larger number of teams to allow for more power and hopefully a significant effect of problem construction on creativity.

While we have speculated that problem construction caused a reduction in conflict and increased satisfaction due to the structure that developed from the process, the exact nature of these relationships is still unclear. Future research should not only replicate the current findings but also add to them by identifying the process by which problem construction operates on these team processes, and whether indeed increased structure is what facilitated the benefits of problem construction. Further, while we expect that on average team composition variables and other relevant variables were equivalent in this sample, due to random assignment, this cannot be fully determined, and should be investigated. It is important to note that effective social processes can be more difficult to attain when teams are diverse (Leung and Wang, 2015). It would be therefore important to study whether the positive effect of problem construction on social processes found here, operates equally on diverse and non-diverse teams.

Another limitation is the use of short-term student teams. While short-term teams exist in organizations, and therefore this research provides meaningful information, the relationships between problem construction and creativity as well as social processes may not operate in the same way in long-term teams in which members share a history and know that they will continue to work with one another. As such, it is important to assess these relationships in long-term teams as well.

To add, although direct information on whether team members have had prior experience with each other was not collected, the current study assumes that the members of the three-person teams were strangers to each other. Given the large size of the university and psychology department and the method used to create groups, we expect that most teams included students that were not familiar with one another. It is possible that some teams had team members that were familiar with each other. In these groups the social processes of conflict reduction and satisfaction may operate differently than in groups composed of strangers. Further research and analyses are need to determine the extent to which this effects influences the conclusions of this study.

Additionally, future research should also seek to reproduce this study’s findings using organizational contexts and samples. Although many of this study’s claims were intended to translate to application in organizational settings, the current study’s findings were derived from data obtained from a student sample, as opposed to employees. Research on the generalizability of undergraduate research participants suggests that university student samples can be used to represent non-student populations when testing psychological processes and behaviors (Lucas, 2003). Despite the testing of such a process, problem construction, the true generalizability of the student sample in this study is unknown and stands as a potential limitation.

Finally, future research should evaluate whether problem construction influences other social process such as psychological safety, trust, or team efficacy. While our choice of studying conflict was a result of past research on rGaps, it is possible that effective problem construction stemming from instructions can also facilitate the development of psychological safety, trust, and communication, contributing to reduce conflict and increase satisfaction.

## Conclusion

This study explores the benefits of problem construction instruction in facilitating creativity in teams. Furthermore, by relating the social process of team satisfaction and conflict to problem construction, this study provides empirical evidence that helps explain the role of team problem construction processes in team productivity. Although much more research is needed, this study contributes an initial look into team level creative cognition using an experimental design. As organizations continue to experience complex problems that surpass an individual’s capacity, a more thorough understanding of the specific components of the creative process including and beyond problem construction at the team level is required.

## Ethics Statement

This study was carried out in accordance with the recommendations of the IRB of the University of Nebraska Medical Center and University of Nebraska at Omaha. The protocol was approved by the IRB. All subjects gave written informed consent in accordance with the Declaration of Helsinki.

## Author Contributions

RR-P was responsible for the conceptualization of the study, data analysis, and writing. VM was responsible for helping with data and writing.

## Conflict of Interest Statement

The authors declare that the research was conducted in the absence of any commercial or financial relationships that could be construed as a potential conflict of interest.
